# Internal Limiting Membrane Flaps for Coexistent Macular Hole and Retinal Detachment in Eyes with Proliferative Diabetic Retinopathy

**DOI:** 10.1155/2018/3470731

**Published:** 2018-11-06

**Authors:** San-Ni Chen, Chung-May Yang

**Affiliations:** ^1^Department of Ophthalmology, Changhua Christian Hospital, Changhua, Taiwan; ^2^Department of Optometry, Da-Yeh University, Changhua, Taiwan; ^3^College of Medicine, Chung-Shan Medical University, Taichung, Taiwan; ^4^Department of Ophthalmology, National Taiwan University Hospital, Taipei, Taiwan; ^5^College of Medicine, National Taiwan University, Taipei, Taiwan

## Abstract

**Purpose:**

To evaluate logical surgical approaches to closing macular holes in eyes with proliferative diabetic retinopathy with retinal detachment.

**Methods:**

Retrospective, interventional case series.

**Results:**

10 eyes in 10 patients were included in this study. The inverted internal limiting membrane (ILM) flap technique was used in 2 eyes, while inverted ILM insertion was used in 5 eyes, and free ILM flaps in 3 eyes. Closed macular holes and retinal reattachment were observed in all eyes. Best corrected visual acuity improved from 1.33 ± 0.39 preoperatively to 1.02 ± 0.36 postoperatively (*p*=0.03).

**Conclusion:**

Various surgical approaches utilized in managing macular holes may effectively close macular holes and reattach retinas. This trial is registered with NCT 03618498.

## 1. Introduction

Macular holes (MH) associated with retinal detachment (RD) most commonly occur in eyes with high myopia. Conventional surgery with vitrectomy and complete internal limiting membrane (ILM) peeling results in an unsatisfactory macular closure rate [1]. Recently, several surgical techniques have been developed, including the use of epiretinal ILM flap covering the hole [2, 3]; ILM flap insertion into the hole, with or without the adjunct of blood clot and viscoelastic agents [4–6]; or even free ILM flaps [7]. High MH closure rates can be achieved. Other than high myopia, MH with RD may appear in several other conditions, such as severe vitreomacular traction, proliferative diabetic retinopathy (PDR) [8, 9], or complicated rhegmatogenous retinal detachment (RRD) from peripheral breaks [10–12].

In eyes of PDR, the combination of MH and RD usually signifies severe and complex vitreoretinal traction; thus, the closure rate of MH after conventional ILM peeling surgery may, as a consequence, also be less satisfactory [8, 9]. Thus, in this retrospective study, we aim to study whether using various techniques including epiretinal ILM flap, ILM insertion, or free ILM flap may effectively close MHs and reattach retinas where MH coexists with RD in PDR eyes.

## 2. Materials and Methods

From September 2014 to January 2018, clinical charts were reviewed of patients with PDR suffering from MH with RD who had been treated with vitrectomy combined with inverted epiretinal ILM flap, inverted ILM flap insertion technique, or free ILM flaps. Two experienced surgeons (SN Chen and CM Yang, from Changhua Christian Hospital and National Taiwan University Hospital, respectively) performed the operations. The study was approved by the ethics committees and research boards of the two hospitals. The severity of RD was separated into 3 types: within the arcade; beyond the arcade but within the equator; and beyond the equator. Active fibrovascular proliferation (FVP) was defined as FVP containing visible neovascularization tissue or associated with any degree of VH; otherwise, it was regarded as “mainly fibrotic.” The extent of FVP was separated into 4 grades based on the severity of vitreoretinal adhesion as previously described [13]: multiple-point adhesions with or without 1 site plaque-like broad adhesion (Grade 1); broad adhesions in more than 1 but fewer than 3 sites, located posterior to the equator (Grade 2); broad adhesions in more than 3 sites, located posterior to the equator or extending beyond the equator within 1 quadrant (Grade 3); and broad adhesions extending beyond the equator into more than 1 quadrant (Grade 4). Distribution of fibrous tissue was separated into 2 types: fibrous tissue along the arcade and fibrous tissue along the arcade and other areas.

Each patient underwent thorough ophthalmological examinations before and after surgery. The patients' demographic data, records of ophthalmological examinations, and surgical procedures were collected, including best corrected visual acuity (BCVA) before and after operation, fundus changes, presence or absence of proliferative vitreoretinopathy, and MH repairing techniques. The macular structure and presence of vertical vitreomacular traction were evaluated via optical coherence tomography, both pre- and postoperatively (Stratus OCT or Cirrus OCT; Carl Zeiss Meditec, Inc, Dublin, CA, or RTVue Premier; Optovue, Inc, Fremont, CA). All patients had a follow-up duration of more than 3 months following reattachment surgery.

### 2.1. Surgical Techniques

Standard 3-port 23- or 25-gauge pars plana vitrectomy was performed. After core vitrectomy, anterior-posterior-oriented traction and all fibrovascular tissues were removed as thoroughly as possible. Any epiretinal membrane causing fixed retinal folds was removed using forceps. The peripheral vitreous was detached as far anteriorly as could be safely done. A 25-gauge blunt-tipped needle connected with a syringe containing Viscoat® (Alcon laboratories, Fort Worth, TX) was placed within the macular hole, just below the level of the macular hole. A small amount of Viscoat® was injected into and around the hole. An ICG solution (25 mg ICG in 15 ml 5% glucose-water solution, final concentration = 1.7 mg/ml) was then carefully applied around the macular hole within the arcade. Excessive ICG was immediately removed via suction. ILM at the parafoveal area was peeled in a circular fashion. Care was taken not to peel the ILM flap across the hole edge. If possible, at least 1.5 to 2 times the disc area of partially detached ILM around the hole was left in place, with the central part remaining attached to the edge of the hole. Further anterior ILM peeling was performed up to the arcade along with the overlying epiretinal membrane (ERM). The pieces of ILM flap removed were saved in a balanced salt solution-soaked sponge for possible later use. The peripheral retina was then examined for possible breaks. Using microforceps, the ILM flap anchoring on the hole edge was inverted and used to cover the hole. If possible, a large semicircular flap (about 180 degrees and 2 disc diameters in size) was created superiorly as previously described [3]. Otherwise, temporal side ILM flap was used. However, if the risk of the ILM flipping back was judged to be high, ILM insertion instead of ILM hole coverage was adopted [5]. If the size of the ILM flaps was judged inadequate, the double ILM insertion technique was used [6], done by adding a piece of previously obtained free ILM flap on top of the inverted ILM tissue until it was securely in place. If, however, the ILM flaps around the hole edge were difficult to create, or ILM tissue adjacent to the hole had been torn away inadvertently or along with ERM, 2 to 3 pieces of free ILM flap were inserted sequentially into the hole instead after copious Viscoat® agent was injected in and around the MH [7]. Fluid-air exchange (FAX) up to the detached margin was performed for localized RD within the arcade. For RD extending to or beyond the equator, a drainage retinotomy was made with the vitreous cutter at the temporal upper detached retina for internal drainage and fluid-air exchange. No complete fluid-air exchange was intended, to avoid disturbance of the inserted ILM flaps. Laser photocoagulation was done around the iatrogenic break. Finally, the air was replaced with a 20% C_3_F_8_ infusion or silicon oil into the vitreous cavity. Patients were kept in a facedown position overnight and were allowed to take any position except supine for approximately one week.

### 2.2. Statistics

The decimal visual acuity was converted to logarithm of a minimal angle of resolution (logMar) for visual acuity (VA). The difference in logMar VA before and after operation was calculated via the Wilcoxon signed-rank test.

## 3. Results

**Figure 1:**
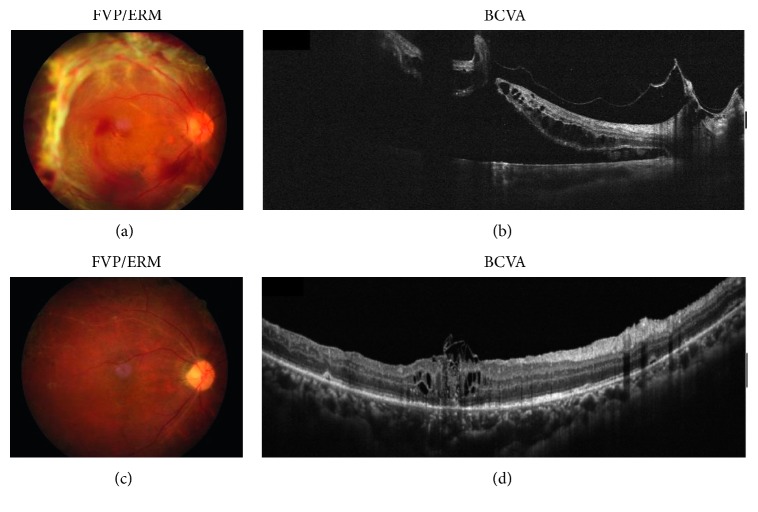
A 33-year-old man with proliferative diabetic retinopathy and active fibrovascular proliferation (case 4) had progressive loss of vision in the right eye. Preoperative fundus (a) and horizontal OCT scan images (b), showing retinal detachment and macular hole. After vitrectomy, internal limiting membrane insertion, air-fluid exchange, and gas tamponade, postoperative fundus (c) and horizontal OCT scan images (d).

There were 10 eyes (M : F = 5 : 5, age: 51.30 ± 10.58 years) included in this study. The demographic data are listed in Table 1. In the 10 eyes, 3 had active FVP and 7 were mainly fibrotic. Minor vitreous hemorrhage which only minimally obscured the posterior fundus view was present in 2 of the 3 eyes with active FVP (cases 4 and 5). All eyes had previous panretinal photocoagulation. Intravitreal injection of bevacizumab 1.25 mg three days before operation was performed in cases of active FVP (cases 3–5). The severity of FVP was grade 2 in the majority. FVP was noted along the arcade in all cases; 4 of the 10 eyes also had ERM across the macula noted. One post-SO removal case also had thickened ERM on the temporal upper and temporal lower arcade. Severity of fibrovascular traction and extent of retinal detachment are listed in Table 1. Only 1 eye had a bullous characteristic (Case 1). OCT showed cystic changes at the MH margin in 9 eyes. Vitreomacular traction on the macular hole was noted in 8 eyes (Figure 1). The average size of MH was 406.50 ± 223.28 microns. Iatrogenic retinal breaks encountered while dissecting FVP occurred in 2 of the 10 eyes, without affecting the final results. The inverted epiretinal ILM flap technique was used in 2 cases; the inverted ILM flap insertion technique (including the double ILM flap technique; see Surgical Techniques) was used in 5 cases (Figure 1); and free ILM flap insertion was used in 3 cases. 20% C_3_F_8_ was infused in 9 eyes and silicon oil in 1 eye as a tamponade at the end of surgery. All eyes had retina reattachment and type 1 MH closure after 1 operation. None of the cases had recurrent vitreous hemorrhage noted during the follow-up period. Demographic data are shown in Figure 1. The best corrected logMar VA improved from 1.33 ± 0.39 preoperatively to 1.02 ± 0.36 postoperatively (*p*=0.03, Wilcoxon signed-rank test).

## 4. Discussion

Multiple traction forces, such as posterior bulging, ERM and ILM tangential traction, or posterior hyaloid oblique traction, are involved in MH with RD in highly myopic eyes. Similarly, multidirectional forces and strong vitreomacular adhesion are required to produce MH with RD in cases of PDR. However, there are some differences in the pathogenesis of MH formation between MH associated with RD in HM and coexisting MH with RD in PDR. In HM, RD generally develops secondary to MH formation. Even after cortical vitreous stripping and ILM peeling, the retinal surface is often insufficient to meet the choroid surface area and allows the hole to close. In contrast, in coexisting MHs with RD in PDR, a long-term vitreomacular traction from VMT or a tangential traction from fibrovascular tissue predisposes MH formation [8, 9] and secondary retinal detachment. In our cases of PDR, the clinical findings suggest that, to induce MH and RD in PDR, the major factors may not be the severity of FVP but the distribution of FVP/fibrotic tissue. All our cases had fibrous tissue distributed at the arcade. The traction force around the arcade that exerts oblique traction on the posterior macula, in addition to the adherent epiretinal membrane usually found in such conditions, may be the primary force that induces RD when an MH forms. These changes create multidirectional traction and testify to the complexity of vitreoretinal relationships.

Although the pathogenesis of MH formation and RD development may be different in highly myopic and PDR patients, both severe traction and tissue degeneration of the fovea are present in both types of cases; thus, a lower rate of MH closure than with idiopathic MH cases is expected after surgical repair with the traditional techniques of membrane peeling and ILM peeling [1, 8]. Uemoto et al. have reported that after ILM peeling, MHs closed in 72.7% of highly myopic eyes with MH-related RD. Yeh et al. have reported that, after release of fibrovascular tissue with or without ILM peeling, MHs were closed in 82.6% of eyes with PDR-related MH [8]. Whether the MH in a specific case will successfully close after conventional surgery, however, has not been predictable. Traction and relative ischemic status predispose toward tissue degeneration of the macula. When severe, complex, long-standing traction and the resultant tissue degeneration are present, macular tissue around the hole might lose its ability to achieve hole closure even after traction force had been released. We postulate the above reasoning as one of the major causes for the unsatisfactory closure rate. In the current study, we offered ILM tissue within or above the macular holes as a bridging tissue in our cases of PDR, as what has recently been adopted in macular holes with high myopia. This technique has proven to be effective in improving the unpredictable MH closure results in coexistent macular hole and retinal detachment in eyes with PDR.

In this series, the initial choice to close the macular hole was always the inverted epiretinal ILM flap technique because this approach interferes least with the photoreceptor regeneration and best avoids possible dye toxicity on the retinal pigment epithelial cells after surgery. Inverted ILM flap insertion or free flaps were used when the size and the integrity of the ILM flap were judged insufficient for adequate MH coverage. When even the ILM insertion, including the addition of a free ILM flap, could not be done, multiple ILM flaps were used. The latter technique did not require a meticulous fovea-spared ILM peeling and was best suited to small MHs [7].

In our case series, only two had inverted epiretinal ILM flaps; others had either inverted ILM insertion (5 cases) or free ILM flaps (3 cases). Because complex traction condition existed in the PDR cases, with multilayered ERM adhered and exerting vertical and/or horizontal traction on the macula, adequately large ILM tissue to cover the MH could not be consistently obtained. Thus, the other techniques were chosen for more secure ILM positioning. Indeed, all 10 of our cases had MH closure with reattached retina.

In our series, only 3 eyes with active fibrovascular proliferation had preoperative intravitreal injection of antivascular endothelial growth factors (anti-VEGFs). Although pretreatment with anti-VEGF agents may reduce bleeding tendency during the operation, it may also aggravate the tractional force of the fibrovascular tissue, and thus it should be judiciously used in eyes with fibrous components.

In this study, we showed that all eyes had type 1 macular hole closure and retina reattachment. Statistically improved visual acuity was also noted. However, the final BCVA was still far less than that observed with idiopathic macular holes. The possible reasons for the limited visual improvement after hole closure may be predisposed disease status, including macular ischemia, cystoid changes secondary to microvascular anomalies in PDR, or schisis changes secondary to long-term traction from the FVP, which irreversibly damage the foveal structure. ICG toxicity to the photoreceptors and the retinal pigment epithelium may also be contributing factors. The limitations of this study are the small group of patients, and the lack of a control group. A larger case number with a proper control study is necessary to clarify whether the closure rate is higher with our present techniques, risk factors for MH closure, and whether closure of macular hole helps further improvement of VA in this difficult group of patients.

## Figures and Tables

**Table 1:** Demographic data of patients.

Case number	Age/sex/eye	MH size	Active/fibrotic	Severity	Distribution	RD extent	Initial	Final	F/U duration	MH closure	Procedure
1	56/M/L	500	F	1	1	3	ND/50 cm	0.03	5M	+	2
2	52/F/R	500	F	2	2	2	0.1	0.05	15M	+	2
3	49/M/R	480	A	1	1	1	0.05	0.05	12M	+	2
4	33/M/R	200	A	2	2	2	0.04	0.25	12M	+	2
5	52/F/L	200	A	3	2	2	0.05	0.05	16M	+	2
6	71/F/R	900	F	1	1	1	0.1	0.1	10M	+	3
7	49/F/L	200	F	2	1	1	0.1	0.2	6M	+	3
8	63/M/L	200	F	2	2	2	0.01	0.05	5M	+	3
9	42/F/L	510	F	2	2	2	0.05	0.2	18M	+	1
10	46/M/L	375	F	2	2	1	0.1	0.3	5M	+	1

BCVA: best corrected decimal visual acuity; F/U: follow-up; MH: macular hole; M: male; F: female; FVP: fibrovascular proliferation; ERM: epiretinal membrane; RD: retinal detachment; L: left; R: right; ND: number of digits. Procedure: (1) inverted internal limiting membrane (ILM) flaps covering macular hole; (2) inverted ILM flaps insertion; (3) free ILM flaps insertion. Severity scale of FVP: Grade 1: multiple-point adhesions with or without 1 site plaque-like broad adhesion; Grade 2: broad adhesions in more than 1 but fewer than 3 sites, located posterior to the equator; Grade 3: broad adhesions in more than 3 sites, located posterior to the equator or extending beyond the equator within 1 quadrant; Grade 4: broad adhesions extending beyond the equator for more than 1 quadrant. Distribution of fibrous tissue: (1) arcade; (2) arcade and other area. RD extent: extent of retinal detachment—(1) within the arcade; (2) beyond the arcade, within the equator; (3) beyond the equator.

## Data Availability

The data used to support the findings of this study are restricted by the name in order to protect patient privacy. Data are available from name for researchers who meet the criteria for access to confidential data.
